# Phylogenetic Analyses of Novel Squamate Adenovirus Sequences in Wild-Caught *Anolis* Lizards

**DOI:** 10.1371/journal.pone.0060977

**Published:** 2013-04-10

**Authors:** Jill M. Ascher, Anthony J. Geneva, Julienne Ng, Jeffrey D. Wyatt, Richard E. Glor

**Affiliations:** 1 Department of Laboratory Animal Medicine, University of Rochester Medical Center, Rochester, New York, United States of America; 2 Department of Biology, University of Rochester, Rochester, New York, United States of America; University of Georgia, United States of America

## Abstract

Adenovirus infection has emerged as a serious threat to the health of captive snakes and lizards (i.e., squamates), but we know relatively little about this virus' range of possible hosts, pathogenicity, modes of transmission, and sources from nature. We report the first case of adenovirus infection in the Iguanidae, a diverse family of lizards that is widely-studied and popular in captivity. We report adenovirus infections from two closely-related species of *Anolis* lizards (anoles) that were recently imported from wild populations in the Dominican Republic to a laboratory colony in the United States. We investigate the evolution of adenoviruses in anoles and other squamates using phylogenetic analyses of adenovirus polymerase gene sequences sampled from *Anolis* and a range of other vertebrate taxa. These phylogenetic analyses reveal that (1) the sequences detected from each species of *Anolis* are novel, and (2) adenoviruses are not necessarily host-specific and do not always follow a co-speciation model under which host and virus phylogenies are perfectly concordant. Together with the fact that the *Anolis* adenovirus sequences reported in our study were detected in animals that became ill and subsequently died shortly after importation while exhibiting clinical signs consistent with acute adenovirus infection, our discoveries suggest the need for renewed attention to biosecurity measures intended to prevent the spread of adenovirus both within and among species of snakes and lizards housed in captivity.

## Introduction

Recent studies identify a growing number of viruses that infect reptiles [1], [2]. The adenoviruses-a group of non-enveloped, double-stranded DNA viruses characterized by intermediate genome size (26–45 KB) and a distinctive icosahedral structure-have emerged as a potentially serious threat to the health of captive reptiles [3]–[7]. Adenoviruses occur as pathogens across vertebrates, including humans, and are widespread among squamate reptiles (i.e., snakes and lizards) (Table 1). Because adenovirus infections can result in significant clinical morbidity and mortality in reptiles adenovirus outbreaks can be devastating for individual animals and to captive colonies [13], [25]–[26].

**Table 1:** Reported squamate species in which adenovirus infections have occurred.

	Species	Clade	Source	Outbreak	Reference
1	*Varanus exanthematicus* (savannah monitor)	Varanidae	RWC^1^	No	[8]
2	*Varanus prasinus* (emerald monitor)	Varanidae	NA^2^	No	[9]
3	*Chameleo jacksoni* (Jackson's chameleon)	Chameleonidae	LTC^3^	Yes(1)	[10]
4	*Chameleo montium* (mountain chameleon)	Chameleonidae	RWC	Yes(1)	[7], [4]
5	*Pogona henrylawsoni* (Rankin's dragon)	Agamidae	LTC	Yes	[11]
6	*Pogona vitticeps* (central bearded dragon)	Agamidae	LTC	Yes	[12], [5], [4], [13] [3]
7	*Pogona minor* (western bearded dragon)	Agamidae	NA	No	[14]
8	*Pogona barbata* (eastern bearded dragon)	Agamidae	NA	No	[15]
9	*Ctenophorus nuchalis* (central netted dragon)	Agamidae	LTC	No	[14]
10	*Heloderma suspectum* (gila monster)	Helodermatidae	LTC?^3a^	Yes	[4], [9]
11	*Heloderma horridum* (Mexican beaded lizard)	Helodermatidae	LTC?	Yes	[9]
12	*Hemitheconyx cuadicinctus* (African fat-tailed gecko)	Gekkonidae	LTC?	Yes	[4]
13	*Eublepharus macularius* (leopard gecko)	Gekkonidae	LTC?	Yes	[4]
14	*Gekko gecko* (tokay gecko)	Gekkonidae	LTC?	No	[4]
15	*Tiliqua scincoides intermedia* (northern blue-tongued skink)	Scincidae	LTC?	No	[4]
16	*Elaphe quatuorlineata* (four-lined rat snake)	Serpentes	RWC	No	[16]
17	*Elaphe longissima* (Aesculapian snake)	Serpentes	RWC	No	[16]
18	*Pantherophis guttatus* (corn snake)	Serpentes	LTC?	No	[17], [18], [19], [20]
19	*Lampropeltis zonata* (mountain kingsnake)	Serpentes	LTC	No	[21], [22]
20	*Lampropeltis getulus californiae* (California kingsnake)	Serpentes	LTC?	NA	[20]
21	*Lampropeltis triangulum* (milksnake)	Serpentes	LTC?	NA	[20]
22	*Boa constrictor (common boa constrictor)*	Serpentes	LTC?	Yes(1)	[16], [6], [9]
23	*Lichanura trivirgata* (rosy boa)	Serpentes	LTC?	No	[23]
24	*Python regius* (royal python)	Serpentes	LTC?	No	[24]
25	*Crotalus scutulatus scutulatus* (Mojave rattlesnake)	Serpentes	NA	No	[6]
26	*Vipera aspis* (asp viper)	Serpentes	NA	No	[9]
27	*Bitis gabonica* (Gaboon viper)	Serpentes	RWC	No	[16]
28	*Parias hageni* (Indonesian pit-viper)	Serpentes	NA	No	[19]
29	*Bothriechis marchi* (palm viper)	Serpentes	NA	No	[21]
30	*Acanthophis antarcticus* (death adder)	Serpentes	NA	NA	Hyndman Genbank submission [14]
31	*Pituophis catenifer sayi* (bull snake)	Serpentes	LTC?	NA	[20]

1 RWC  =  Recently wild caught, ^2^NA  =  information on source not available, ^3^LTC  =  long-term captive (possibly including individuals that were born and bred in captivity), ^3a^LTC?  =  likely long-term captive, but no information on origins in original publication.

**Figure 1:**
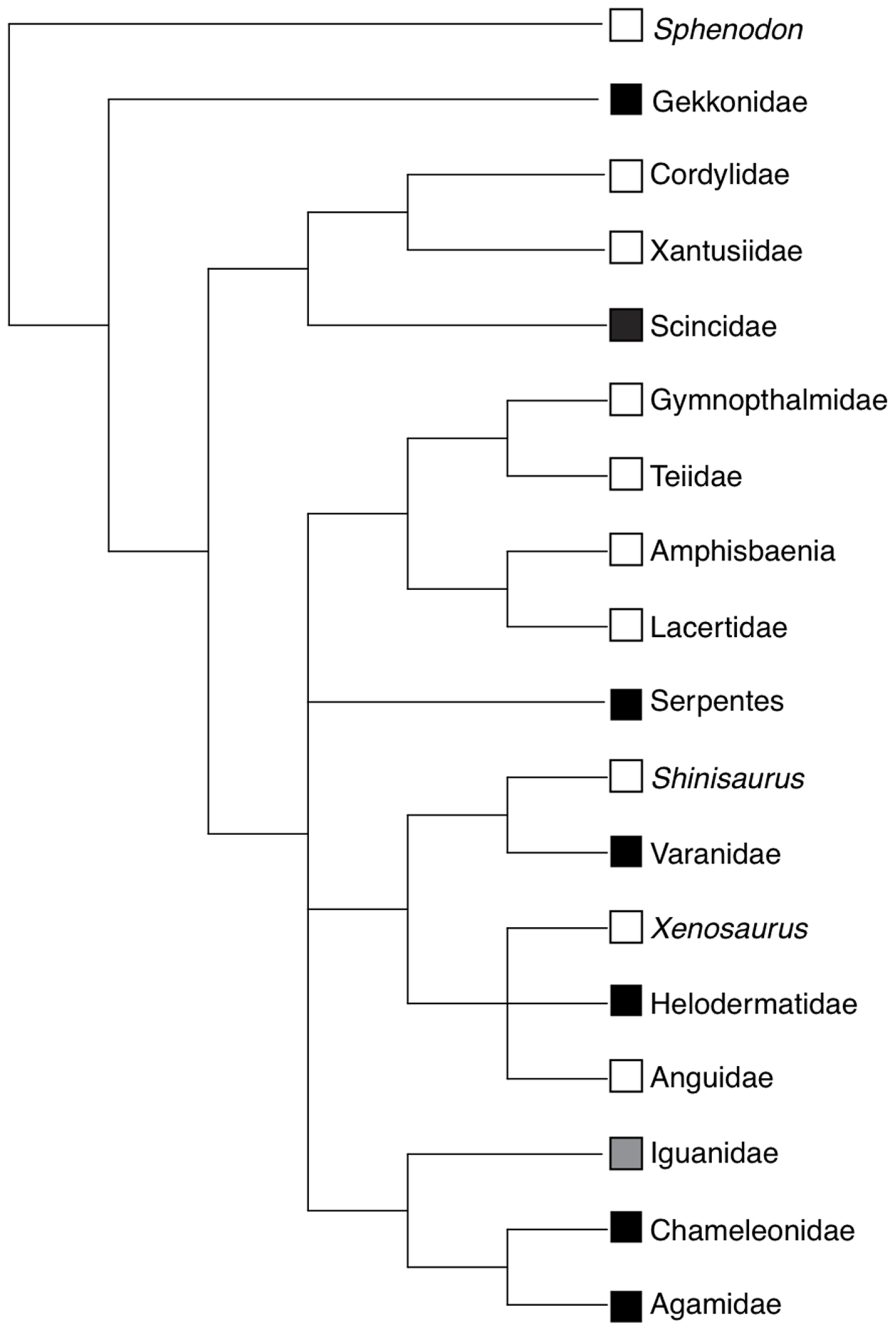
Phylogenetic tree for squamate reptiles derived from Townsend et al. (2004). Black squares indicate clades with previously reported adenovirus infections, white squares indicate clades without previously reported infections, and the gray square indicates the clade from which adenovirus infection is reported in this study.

Case reports of reptiles infected with adenovirus have been on the rise in recent years and now include 28 species sampled from across the squamate phylogenetic tree (Fig. 1, Table 1). However, most of these infections are reported from captive animals and the list of afflicted species reads like a who's who of the species most popular among reptile hobbyists. Indeed, most recent adenovirus infection reports involve one of the most popular pet shop lizards in the United States, the central bearded dragon (*Pogonavitticeps*). Although adenoviruses now appear enzootic to captive populations of bearded dragons and other popular captive reptiles, we know relatively little about the extent of their phylogenetic distribution across the host clade, pathogenicity, modes of transmission, and sources in nature.

As reports of adenoviruses from squamates in nature are apparently non-existent, we are particularly ignorant of the likely sources for squamate adenovirus infections in captive lizards. Most of the reported infections are from long-term captives, including many individuals that have likely been in captivity for generations (Table 1). Some infections have been described from recently imported animals, but the precise sources of these animals are never reported and many have been housed with other species at some point in their captive histories. Although their complexity, large size, and comparatively slow rate of evolution relative to small DNA viruses are often cited to support models involving host-specificity and co-speciation in adenoviruses [4,9,27,28], several lines of evidence suggest that occasional host transfers do occur. First, phylogenetic studies hypothesize multiple ancient host transfers of adenoviruses belonging to the genus *Atadenovirus* from reptiles to birds, marsupial mammals and ruminant mammals [18], [19]. Second, recent studies recover identical or nearly identical adenovirus sequences from deeply divergent reptile species, suggesting recent host transfer events among captive animals [14], [29]; one adenovirus sequence has been isolated from three distantly related snake species and a second sequence has been isolated from three distantly related squamate clades (helodermatids, agamids, and serpents) [14], [29].

Here we report discovery of two new adenovirus sequences from a group of lizards that is conspicuously absent from the list of taxa with known infections: the Iguanidae [30]. The absence of reported iguanid adenovirus infections is surprising because the group includes the green iguana (*Iguana iguana*), rock iguanas (*Cyclura*), anoles (*Anolis*) and many other species that are popular among reptile hobbyists. We recovered new adenovirus sequences from two closely-related subspecies of *Anolis* lizards that were recently imported from the Dominican Republic: *A. distichus ignigularis* and *A. d. ravitergum* (both of which may actually represent distinct species) [31]. We collected these animals from natural populations and accompanied them throughout the importation process, ensuring that they had no contact with other captive squamates prior to arrival in the laboratory colony. Moreover, all of the infected animals were housed in a facility that has only ever housed anoles and related new world iguanids (e.g., *Polychrus*).

We use sequence data from the adenovirus polymerase gene to test two predictions of the hypothesis that adenoviruses are host specific and strictly co-speciating with their hosts: (1) the two anole species in our study will be infected by novel specific adenovirus sequences endemic to natural populations, and (2) anole adenoviruses will be recovered as sister to adenoviruses sampled from agamids and chameleons, which together form a group known as the Acrodonta that is well-supported as the sister group to Iguanidae (together the Acrodonta and the Iguanidae form the Iguania, see Fig. 1).

## Materials and Methods

### Ethics Statement

Our institutional IACUC, The University of Rochester University Committee on Animal Resources approved this study. The Ministerio de Medio Ambiente y Recursos Naturales in the Dominican Republic provided permission to conduct our field sampling.

### Sampling

**Figure 2:**
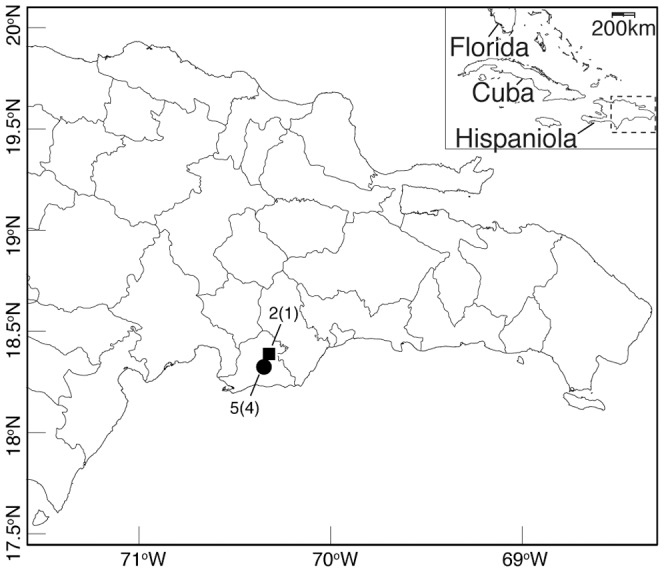
Collecting locations of *A. d. ignigularis* (square) and *A. d. ravitergum* (circle) near Baní, Dominican Republic. Numbers reflect sample size of symptomatic lizards tested for adenovirus and the number of positive tests in parentheses.

In January 2011, we imported 320 lizards representing 40 mature males and 120 mature females from each of two species of *Anolis* lizards, *A. d. ignigularis* and *A. d. ravitergum*, from two different sites in the Dominican Republic (Fig. 2). All *A. d. ignigularis* were collected from a single site in a mesic broadleaf forest adjacent to the Rio Baní while all *A. d. ravitergum* were collected from a second site in an avocado plantation surrounded largely by xeric scrub forest approximately 6 km away. These animals were imported in insulated containers with supplemental heating packs, and shipped as airplane cargo to an AAALAC-accredited facility located in Rochester, NY. Anoles were maintained in this facility and provided with food, housing, lighting, and environmental parameters according to established standard operation procedures for these species, and approved by the University of Rochester Institutional Animal Care and Use Committee (Rochester, NY). The room temperature and photoperiod are cycled to mimic natural seasonality. All anoles are kept in commercially available reptile enclosures (Lee's Kritter Keeper, L Schultz, San Marcos, CA) in breeding groups of 1 male and 2 females. Each cage includes potting soil for substrate, perches and artificial foliage. Each cage is misted twice daily to supply anoles with drinking water. Anoles are fed *ad libitum* two or three times weekly depending on the season. At every feeding, crickets are dusted with a multivitamin supplement (Herptivite, Rep-Cal, Los Gatos, CA) and once weekly with a 1∶1 mix of the multivitamin supplement and a calcium additive (Calcium Powder, Rep-Cal) [32].

Approximately three weeks after arrival at our laboratory colony, which also houses other *Anolis* lizards acquired during previous field sampling, some of the newly imported lizards began to exhibit non-specific clinical signs of illness. Only females from this recent shipment became ill, with the illness presenting in waves from February 21, 2011 to March 31, 2011. Eventually, 31 of 320 female lizards were afflicted. The illness was first identified in lizards that exhibited significant weight loss. Upon closer examination, these lizards were found to be lethargic, with poor appetites progressing to anorexia. In addition, animals with clinical signs were consistently observed at the bottom of the cage with their eyes closed. The affected females appeared smaller than the average sexually mature female *Anolis* lizard, although no quantitative measurements were taken in order to confirm this observation. Supportive care was administered to all affected animals including hand-feeding of turkey baby food for protein, mixed with water to help support hydration. To reduce infection between cages, sick animals were treated and cared for only after all other cages had received husbandry. A single laboratory member administered husbandry and treatment to the clinically affected lizards, wearing gloves and washing her hands afterwards. Additionally, lizards displaying clinical signs were isolated from healthy lizards, and had no contact with the other lizards in the facility. To date, none of the other established colony resident animals have presented with similar clinical signs. A representative female anole (*Anolis distichus ravitergum*) exhibiting the signs described above was euthanized humanely with sodium pentobarbital, per an approved method listed in the protocol. There were no gross lesions and no other concurrent infections identified in this animal. The animal was necropsied, and multiple tissues were collected, including tissues from the thoracic cavity and coelomic cavity. The submitted tissues were preserved in 10% buffered formalin, and sent to Northwest ZooPath, Monroe, Washington for diagnosis by histopathology. This histopathological work recovered moderate, subacute to chronic gastroenterocolitis with adenovirus-like inclusions, suggestive of infection with adenovirus. This motivated us to use molecular techniques to further characterize the presence of adenovirus in captive *Anolis*.

### Virus sequencing and phylogenetic analyses

We used a nested PCR with consensus primers to test for the presence of adenovirus infection in anoles from the same shipment and subsequently sequenced these PCR products. To obtain tissue samples for the PCR assays, we used sterile, disposable razor blades to subsample approximately 20 mg of abdominal tissue that included liver, stomach and intestinal tissues from two *A. d. ignigularis* and five *A. d. ravitergum* females that died after becoming ill shortly after importation. We extracted DNA from all samples using a Wizard DNA extraction kit (Promega, Madison, WI) following the manufacturer's procedure.

As a control, we determined whether intact DNA was present in each extraction by amplifying a segment of the host specimen's mitochondrial genome using PCR. We attempted amplification of an approximately 1200 base pair fragment of mtDNA that included complete sequence for the genes encoding ND2, tRNA^Trp^ and tRNA^Ala^. We performed PCR amplification using primers located in tRNA^Met^-L4437 [33] and tRNA^Asn^-H5934 [34]. PCR reactions were performed at a total volume of 25 µl with 10.375 µl deionized, reverse osmosis filtered water, 2.5 µl each of forward and reverse primer at 2 µM concentration; 2.5 µl 10× magnesium free Taq reaction buffer; 2.5 µl MgSO_4_ (20 µM); 2.5 µl dNTP mix (5 µM); 0.125 µl Taq DNA Polymerase (5 µ/ µl); and 2 µl of genomic template DNA. Taq DNA polymerase, dNTPs, and 10× buffer were all supplied by Bio Basic Inc. (Markham, ON, Canada). Thermocycling conditions for ND2 PCR began with an initial denaturation at 94°C for 120 seconds followed by 30 cycles of denaturing at 94°C for 35 seconds, annealing at 52°C for 35 seconds, and extension at 72°C for 90 seconds, followed by a final extension at 72°C for 10 minutes. We excluded any samples that failed to amplify ND2.

To screen for the presence of adenovirus DNA, we used previously published nested primers to amplify a highly conserved approximately 320 bp fragment of adenoviral DNA polymerase gene [4]. We chose to amplify this region rather than other regions such as hexon, because the polymerase sequence is available from a larger number of previously reported adenovirus sequences due to the availability and widespread use of primers for the polymerase gene. We performed initial amplifications with external primers (polF-outer and polR-outer) and subsequently used 2 µL of the resulting PCR product as template for a second set of reactions using internal primers (polF-inner and polR-inner). Thermocycling conditions for both reactions began with an initial denaturation at 94°C for 720 seconds followed by 45 cycles of denaturing at 94°C for 30 seconds, annealing at 46°C for 60 seconds, and extension at 72°C for 60 seconds, followed by a final extension at 72°C for 10 minutes. Reagents (excluding primers) and reaction volumes for adenovirus amplifications were identical to those used for mitochondrial amplifications.

We visualized PCR products by gel electrophoresis on 1.5% agarose gels stained with SYBR Safe (Life Technologies, Grand Island, NY). Successfully amplified adenovirus PCR products were purified using ExoSAP (USB Corp., Santa Clara, CA), and sequenced in both directions using the Big Dye Terminator v3.1 system (Life Technologies, Grand Island, NY) on an ABI PRISM 3730xl capillary sequencer (Life Technologies, Grand Island, NY) at the University of Rochester's Functional Genomics Center. We assembled, inspected, and edited nucleotide sequences using Geneious v5 [35]. We aligned sequences by eye and alignment was unambiguous.

We translated nucleotide sequences to amino acids (AA), because of the previously reported AT richness and codon biases among some *Atadenovirus* sequences [36]. We aligned the AA sequences derived from anoles to previously published adenoviral polymerase sequences, including all reported *Atadenovirus* sequences and a broad sampling of sequences representing the remaining four adenovirus genera, which are reported from other vertebrate clades (Table 2).

**Table 2:** Adenovirus sequences analyzed.

Sequence	Genbank	Host Species
Anolis AdV-1	KC544015	*Anolis distichus ignigularis*
Anolis AdV-2	KC544016	*Anolis distichus ravitergum*
Human AdV-12	AY780216	*Homo sapiens*
Human AdV-12	M14785	*Homo sapiens*
Agamid AdV-1 isolate 5	ACH86250	Pogona vitticeps
Agamid AdV-1 strain A1	AAS89694	Pogona vitticeps
Agamid AdV-1 strain C1	ACI28428	Pogona vitticeps
Bat AdV-2 strain PPV1	JN252129	Pipistrellus pipistrellus
Bat AdV-isolate 1069	GU226963	Myotis ricketti
Bat AdV-isolate 1213	GU226951	Myotis ricketti
Bat AdV-isolate 1282	GU226960	Myotis ricketti
Bat AdV-isolate 1391	GU226964	Myotis ricketti
Bat AdV-isolate 1497	GU226967	Myotis ricketti
Bovine AdV-3	AF030154	Bos taurus
Bovine AdV-4	NC_002685	Bos taurus
Box turtle AdV-1	EU828750	Terrapene ornata
Canine AdV-1	Y07760	Canis familiaris
Canine AdV-2	U77082	Canis familiaris
Chameleon AdV-1	AY576679	Chameleo montium
Duck AdV-1	NP_044702	Ducks, geese, chickens
Eublepharid AdV-1	AY576677	Hemitheconyx caudicinctus
Fowl AdV-5	DQ159938	*Gallus gallus*
Frog AdV-1	NC 002501	*Rana pipiens*
Gekkonid AdV-1	AY576681	*Gekko gecko*
Great tit AdV-1	FJ849795	*Parus major*
Helodermatid AdV-1	AY576680	*Heloderma suspectum*
Helodermatid AdV-2	ACH86252	*Heloderma horridum*
Human AdV-11a	FJ597732	*Homo sapiens*
Human AdV-B strain Guangzhou01	DQ099432	*Homo sapiens*
Human AdV-16	AY601636	*Homo sapiens*
Human AdV-3	AY599836	*Homo sapiens*
Human AdV-7	AY601634	*Homo sapiens*
Meyers parrot AdV-1	AY644731	*Poicephalus meyeri*
Bovine AdV-2	AC_000001	Ovis aries
Ovine AdV-7	OAU40839	Ovis aries
Plum headed parakeet AdV-1	EU056825	Psittacula cyanocephala
Porcine AdV-5	AF289262	Sus scrofa domesticus
Pygmy Marmoset AdV	HM245776	Callithrix pygmaea
Scincid AdV-1	AAS89698	Tiliqua scincoides intermedia
Simian AdV-33	FJ025908	*Pan troglodytes*
Simian AdV-35.1	FJ025912	*Pan troglodytes*
Snake AdV-1	NC009989	Pantherophis guttatus
Snake AdV-2	FJ012163	Lampropeltis getula californiae
Snake AdV-3	ACH91015	Pituophis catenifer
Snake AdV-strain GER09	ADT91320	Pantherophis guttatus
Sulawesi tortoise AdV-1	EU056826	Indotestudo forsteni
Tree shrew AdV-1	AF258784	*Tupaia sp.*
Varanid AdV	ACH86253	Varanus prasinus
White Sturgeon AdV	AY082701	Acipenser transmontanus

We used ProtTest v3 [37] to infer an appropriate evolutionary model of amino acid substitution for the final amino acid alignment. Using this model, we inferred relationships among adenovirus polymerase sequences using the Bayesian Metropolis-coupled Markov Chain Monte Carlo (MC3) algorithm in MrBayes v3.1.2 [38]. We ran two independent Metropolis-coupled Markov Chain Monte Carlo runs for 10^8^ generations, each with one cold and three heated chains. We assessed the convergence of Bayesian analyses using three strategies. First, using custom scripts in R v2.14.1 [39] we evaluated model likelihood and Average Standard Deviation of Split Frequencies (ASDSF) for stationarity. Second, we evaluated bipartitions for stationarity and compared support for bipartitions in the two independent runs, using the “cumulative” and “compare” utilities in Are We There Yet? [40]. Finally, we visually inspected the posterior distributions of the model parameters estimated during the MC3 runs and confirmed that these values reached stationary distribution and had sufficiently large effective sample sizes using Tracer v1.5 [41].

To assess the prediction that atadenoviruses co-speciate with their squamate hosts we performed a Shimodaira-Hasegawa (SH) test [42]. We first pruned the consensus tree recovered by MrBayes from adenovirus sequences to include only squamate hosts. We then created a modified version of this tree to match the most current phylogeny of squamate reptiles (Fig 1) [43]. We performed a SH test with 1 million bootstrap replicates using the R package Phangorn [44] to evaluate the fit of this squamate phylogeny to the adenovirus sequences relative to the topology inferred by MrBayes.

## Results

### Virus sequencing and phylogenetic analyses

After excluding one *A. d. ravitergum* individual from our adenovirus screen due to a failure to amplify ND2, we recovered adenovirus DNA from five of the six remaining abdominal tissue samples from sick lizards (including all four *A. d. ravitergum* and one of the two *A. d. ignigularis*). We recovered adenovirus DNA fragments that were either 89 or 92 AA long from each of the five adenovirus-positive samples (this difference in sequence fragment length results from trimming of low quality terminal nucleotides rather than insertion or deletion). All *A. d. ravitergum* sequences were identical in nucleotide sequence while the single *A. d. ignigularis* sequence differed from the *A. d. ravitergum* sequences at 45 nucleotide sites representing nine amino acid substitutions. AT content was 51.9% and 47.6% for *A. d. ignigularis* and *A. d. ravitergum* adenovirus sequences, respectively, similar to AT richness observed in other squamate *Atadenovirus* sequences (AT 41.1–56.3%), and in contrast to the more extreme AT bias observed in *Atadenovirus* sequences reported from mammals and birds [36].

We aligned and trimmed sequences from public databases to match the *Anolis* sequences. We translated nucleotide sequences to amino acids and removed identical sequences from the dataset prior to phylogenetic analyses, leaving a final set of 49 unique sequences (Table 2). ProtTest identified the JTT model with a Gamma distribution of rate categories as the optimal model of molecular evolution for our dataset.

**Figure 3:**
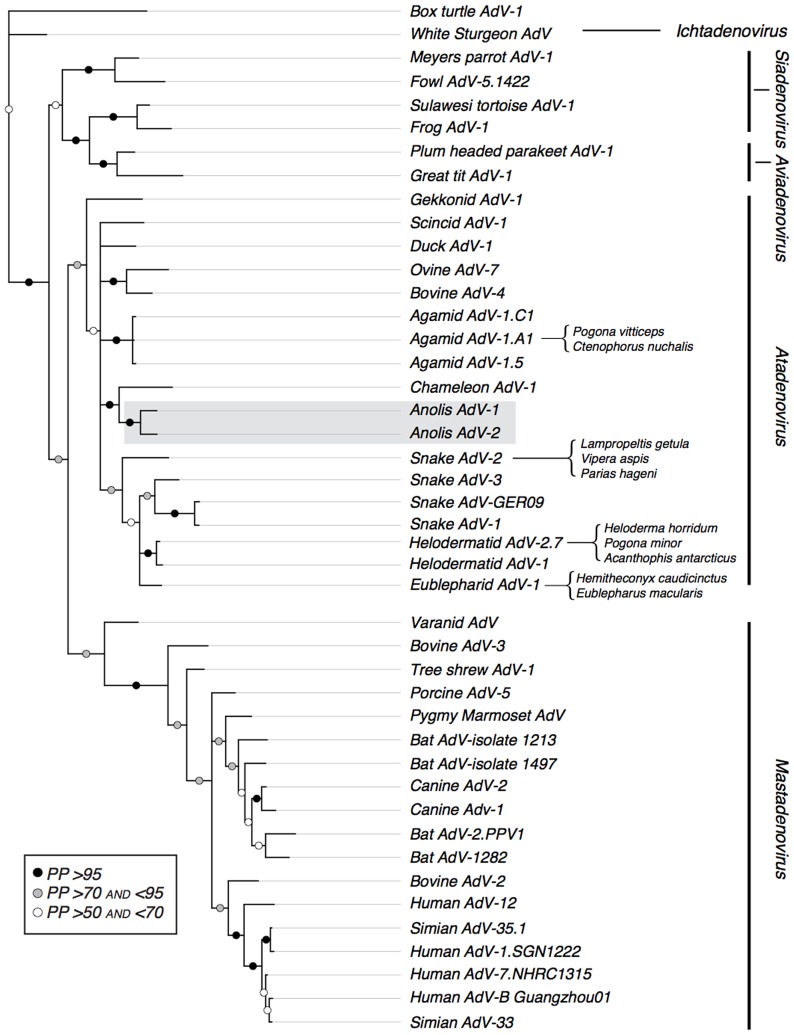
Phylogenetic relationships among adenovirus polymerase sequences inferred using the amino acid models implemented in MrBayes. The phylogenetic position of newly acquired sequences is indicated in grey. Node support values presented are posterior probabilities (PP): black circles PP>95, grey circles 95>PP>70, and white circles 70>PP>50. To simplify the graphical representation of this result, we pruned from the tree adenoviruses that were drawn from the same host and were strongly supported as monophyletic. When a sequence has been reported from two or more reptilian species, we indicate the infected species with a bracket.

All convergence diagnostics suggest that all MC3 analyses in MrBayes converged by 5×10^7^ generations. We discarded these as burn-in and generated a consensus topology from the remaining trees in the posterior distribution using the sumt command in MrBayes. This topology (Fig. 3) is largely concordant with previous analyses and recovers the five recognized adenovirus genera–*Ichtadenovirus*, *Siadenovirus*, *Aviadenovirus*, *Atadenovirus* and *Mastadenovirus*–as reciprocally monophyletic, although relationships among these genera are weakly supported. Concordant with a previous distance based analysis, Varanid adenovirus is found to be outside all five major clades [9], and is instead recovered as sister to the *Mastadenovirus* clade with moderate support. Within the genus *Atadenovirus*, a single gekkonid sequence is recovered as the outgroup to a poorly supported clade with a large basal polytomy that includes atadenovirus sequences sampled from birds, mammals and other squamates. Although sequences sampled from both mammals and birds appear to render the squamate atadenoviruses non-monophyletic, the node supporting nestedness of avian and mammalian adenoviruses within the squamate atadenoviruses is not well supported (posterior probability <70%) (Fig. 3).

Phylogenetic relationships among squamate atadenoviruses do not reflect phylogenetic relationships among host species. When compared to the topology inferred by MrBayes a Shimodaira-Hasegawa test significantly rejects a host-sequence co-speciation tree topology (p<0.000001). The novel *Anolis* sequences detected here are sister to a sequence previously reported from chameleons, rather than being sister to the clade that includes sequences reported previously from Acrodonta (agamids plus chameleons). Three agamid adenoviruses sampled from *Pogona vitticeps* and closely related species form a well-supported clade that descends from a polytomy that also gave rise to atadenoviruses sampled from snakes, geckos, helodermatids and other agamids. The fourth agamid adenovirus sequence, meanwhile, is nearly identical to a sequence also sampled in helodermatids. One gekkonid atadenovirus is weakly supported as the sister to all other atadenoviruses (including avian and mammalian samples) and the second appears closely related to the atadenovirus sequences sampled from snakes and helodermatids. *Anolis distichus ignigularis* and *A. d. ravitergum* derived sequences are recovered as sister lineages nested within other known atadenoviruses. Among the adenoviruses sampled, the *Anolis* adenoviruses are most closely related to the adenovirus derived from a wild-caught chameleon [4]. Finally, support for ancient transfer of *Atadenovirus* to birds and mammals hypothesized in previous reports [18], [19] is equivocal in our study, because the nodes that nest the avian and mammalian adenovirus sequences in the squamate sequences are poorly supported.

## Discussion

We report the first evidence for adenovirus infection in iguanid lizards. Using DNA sequences, we confirmed adenovirus infection in five individuals representing two closely related anole species (*Anolis distichus ignigularis* and *A. d. ravitergum*). These individuals were among 31 females that became gravely ill several weeks after import of 320 individuals from two wild populations in the Dominican Republic. Because we collected these animals from natural populations and accompanied them throughout importation, we can be certain that they were not in contact with other squamate species prior to arrival in the laboratory colony. Moreover, the laboratory colony housing these animals has only ever housed anoles and related iguanids where no previous outbreaks including similar clinical signs have been observed. We therefore hypothesize that the novel adenovirus sequences recovered during our study are enzootic to *Anolis* populations in nature. Of course, until the sequences corresponding to these adenovirus sequences are recovered from animals in nature, we cannot rule out the possibility that adenovirus invaded the facility through some other mechanism (e.g., via feeder crickets obtained from a commercial supplier, on cage dressings, or via a human intermediary). Alternatively, animals stressed by recent capture and transport could have fallen victim to adenovirus strains enzootic in our existing colony. In this case, newly imported animals exposed for the first time to these novel adenoviruses would have had no natural immunity to these viruses and would have been more likely to succumb to illness due to these particular adenovirus strains. Newly imported animals were kept separate from the existing colony, which could have helped to limit the transfer of adenovirus from existing to newly imported animals. However, the same laboratory member cared for both existing and newly imported animals, and cross-contamination was possible. Additionally, imported animals may have been latently infected with an enzootic adenovirus strain. If so, these lizards may have been stressed sufficiently due to recent transport that they succumbed to native adenovirus strains, of which they had been previously tolerant. Finally, although adenoviruses are notoriously environmentally stable, they could not have survived in the potting soil used as cage substrate because this is autoclaved prior to use.

Supporting our prediction that the two adenoviruses identified in this study are novel and host-specific, we recover previously unidentified adenovirus sequences from each of the two closely-related species in our study (Fig. 3). Although somewhat divergent from one another, the two *Anolis* adenovirus sequences are clearly more similar to one another than they are to any other previously reported adenovirus sequences (Fig. 3). Our phylogenetic analyses, however, challenge the predictions of a co-speciation model, under which anole-associated adenovirus sequences should be sister to a clade consisting of adenoviruses sampled from both chameleons and agamids. We found instead that the anole sequences are well-supported as the sister clade to an adenovirus sequence sampled from a chameleon, but the agamid adenovirus sequences occur elsewhere in the phylogeny (Fig. 3). Perhaps not coincidentally, the chameleon adenovirus sequence is among the other sampled sequences that is most likely to have been derived directly from natural populations; the individuals from which this sequence was reported were recently imported from nature, and maintained in strict quarantine prior to the onset of adenovirus associated illness [7].

Agamid-associated adenovirus sequences appear in two places in our phylogenetic tree (Fig. 3), and the origin of these adenovirus sequences is considerably less certain than the origin of adenovirus sequences sampled from anoles and the chameleon. *Pogona* has been widely propagated in captivity for generations in its native Australia and by hobbyists in Europe and the United States, making it difficult to determine whether this adenovirus sequence was carried from *Pogona* populations in nature or results from horizontal transfer following exposure to adenoviruses enzootic to other species of captive reptiles. Although sampling of adenoviruses from *Pogona* in nature are required to test this hypothesis, the available sampling from captive squamates suggests several likely instances of horizontal transfer among distantly related squamates. In one case, Hyndman and Shilton (2011) recovered an adenovirus sequence from an agamid host that was previously found associated with helodermatid lizards [14]. The infected helodermatids were long-term captives in a Danish zoo while the agamid and the snake were captive in their native Australia. Together with our phylogenetic results, these findings suggest that lizard adenoviruses are not necessarily host-specific and do not exclusively co-evolve with their lizard hosts.

More work is now needed to assess the pathology of the adenovirus sequences identified in our study. Consistent with previous reports of acute illness associated with adenovirus infection, all of the sick animals from which we recovered adenovirus via PCR were likely weakened by stress associated with importation or reproduction. Moreover, we do not know whether the adenovirus was the causal factor responsible for illness or merely a secondary, opportunistic pathogen, as a result of a distinct primary cause of illness in the recently transported anoles. Although our study suggests that adenovirus is enzootic to wild populations of *Anolis*, other animals have remained healthy for a year or more following importation to our captive colony, suggesting that some animals may be asymptomatic carriers.

## Conclusions

Our study expands the phylogenetic distribution of squamate adenovirus hosts to include iguanid lizards and finds that adenovirus infection in *Anolis* may be associated with significant mortality of female lizards that have been recently imported from the wild. Furthermore, our phylogenetic analyses of new adenovirus sequences detected in anoles together with adenovirus sequences reported from other squamates further challenge the hypothesis that adenoviruses are host specific and evolving via co-speciation with their hosts. These discoveries should motivate renewed attention to biosecurity measures in captive reptile colonies that are intended to prevent the spread of adenovirus both within and among species [27].
